# Influence of Oxidative and Hydrothermal Pre-Treatments on KOH Activation of Coconut Fiber for Enhanced Supercapacitor Performance

**DOI:** 10.3390/ma19132797

**Published:** 2026-07-01

**Authors:** Eduardo Tovar-Martínez, Isabel Pereyra, Miguel Ángel González-López, María Guadalupe Navarro-Rojero, Jan Mayen, Mayra del Ángel-Monroy

**Affiliations:** 1 CIATEQ A.C., Eje 126 No. 225, Zona Industrial del Potosí, San Luis Potosí C.P. 78395, Mexico; eduardo.tovar@ciateq.mx (E.T.-M.); isabel.pereyra@ciateq.mx (I.P.); mangel.gonzalez@ciateq.mx (M.Á.G.-L.); 2 CIATEQ A.C., Av. del Retablo 150, Constituyentes FOVISSSTE, Santiago de Querétaro C.P. 76150, Mexico; maria.navarro@ciateq.mx; 3 SECIHTI–CIATEQ A.C., Eje 126 No. 225, Zona Industrial del Potosí, San Luis Potosí C.P. 78395, Mexico

**Keywords:** activated carbon, coconut fiber, supercapacitors, KOH activation, hydrothermal treatment, pseudocapacitance, biomass-derived carbon

## Abstract

**Highlights:**

- Pre-treatment strongly influences structure and electrochemical performance of ACs.
- Hydrothermal route yields highest capacitance and superior rate capability.
- Oxidative treatment enhances pseudocapacitive (diffusion-controlled) contribution.
- Charge storage governed by balance between EDLC and pseudocapacitance.
- Coconut fiber is a viable low-cost precursor for high-performance supercapacitors.

**Abstract:**

The development of sustainable electrode materials for supercapacitors requires a deeper understanding of the relationship between precursor structure, processing, and electrochemical performance. In this work, coconut-fiber-derived activated carbons were synthesized via KOH activation, and the influence of oxidative and hydrothermal pre-treatments was systematically investigated. The materials were characterized by X-ray diffraction (XRD), Raman spectroscopy, and Fourier transform infrared spectroscopy (FTIR), while electrochemical performance was evaluated using cyclic voltammetry and galvanostatic charge–discharge measurements in a three-electrode system with 1 M H_2_SO_4_ electrolyte. The results show that hydrothermal pre-treatment leads to improved electrochemical performance, with CF-HTC-AC exhibiting a specific capacitance of ~332 F g^−1^ at 0.5 A g^−1^ and enhanced rate capability. In contrast, the oxidatively treated sample (CF-OC-AC) presents a higher diffusion-controlled contribution, indicating a stronger pseudocapacitive behavior associated with oxygen-containing functional groups. These findings demonstrate that electrochemical performance is governed by a balance between capacitive and diffusion-controlled processes rather than by a single structural parameter. The hydrothermal pre-treatment provides an effective strategy to optimize this balance, highlighting precursor conditioning as a key factor in the design of biomass-derived carbon electrodes for supercapacitor applications.

## 1. Introduction

The rapid electrification of transportation, proliferation of portable electronics, and large-scale integration of intermittent renewable resources have intensified the demand for electrochemical energy-storage systems that can operate efficiently across a wide range of time scales. In this landscape, batteries and supercapacitors are often viewed as complementary rather than competing technologies. Batteries are generally favored when high energy density is required, whereas supercapacitors are preferred for applications demanding fast charge–discharge response, high power delivery, and long operational lifetime [1,2]. The continued expansion of renewable energy systems therefore strengthens interest in electrode materials that can narrow the energy-density gap of supercapacitors without sacrificing their intrinsic advantages in power capability and cycling durability [1,3].

Supercapacitors store charge through two principal mechanisms. In electric double-layer capacitors, energy is stored by reversible electrostatic adsorption of electrolyte ions at the electrode–electrolyte interface, making the accessible surface area and pore architecture of the carbon electrode decisive for performance [4,5]. In pseudocapacitive systems, charge storage additionally involves fast, and reversible surface or near-surface redox reactions associated with electroactive functionalities or heteroatom-containing species, which can enhance capacitance beyond a purely electrostatic contribution [5]. The practical value of a supercapacitor electrode is therefore judged not by capacitance alone but also by a coupled set of figures of merit that includes specific capacitance, energy density, power density, rate capability, and cycle stability. These metrics are strongly governed by ion accessibility, pore connectivity, internal resistance, and the degree to which the electrode structure remains stable during repeated cycling [4,6].

Among the large family of electrode materials investigated for supercapacitors, carbon-based materials remain dominant because they combine chemical robustness with comparatively low cost, high electrical conductivity, and broad tunability of texture and surface chemistry [1,2,7]. Activated carbons are particularly attractive because activation can generate very high specific surface area together with tailored distributions of micro- and mesopores. Micropores contribute substantially to charge storage by increasing ion-accessible interfacial area, while mesopores and larger transport channels facilitate electrolyte diffusion and reduce kinetic limitations, especially at high current density [5,6,8]. As a result, the best-performing activated carbons are rarely those with the highest surface area alone; rather, they are those in which surface area, pore size distribution, and conductive pathways are balanced to support both charge accumulation and rapid ion transport [1,4,9].

Within this broader carbon platform, biomass-derived carbons have attracted growing attention as sustainable electrode materials because they can transform low-value agricultural residues into functional porous carbons through relatively simple thermochemical routes [2,3,10,11]. This approach aligns well with circular economy principles by reducing waste burden, lowering precursor cost, and decreasing reliance on fossil-derived feedstocks. The structural and chemical diversity of biomass also offers a versatile starting point for tuning carbon yield, pore development, and surface functionality, all of which are central to supercapacitor performance [1,2,12].

Coconut fiber is a particularly compelling precursor in this context. As an abundant lignocellulosic by-product of coconut processing, it is widely available in many tropical regions and is often underutilized despite its substantial valorization potential [13,14]. Its intrinsic fibrous morphology and lignocellulosic composition provide a favorable framework for carbonization and subsequent pore development, enabling the production of lightweight porous carbons with interconnected structures [13]. Recent studies have shown that coconut-fiber- and coconut-husk-derived carbons can deliver competitive electrochemical performance when their pore architecture is properly engineered, including hierarchical porous activated carbons with high-rate capability [13], coir-pith-derived activated carbons with high capacitance and good cycling retention [14], and mesoporous carbons obtained through hydrothermal and chemical activation strategies [15,16]. These results confirm that coconut-derived waste is not merely a sustainable precursor, but a technically relevant platform for advanced supercapacitor electrodes.

The electrochemical behavior of coconut-fiber-derived activated carbon depends strongly on the activation route used to create and refine its porous framework. Physical activation, typically using CO_2_ or steam, is attractive because it avoids corrosive activating salts and can produce porous carbons through comparatively simple and greener processing, although pore development may be less aggressive and more difficult to optimize for very high capacitance [17,18]. Chemical activation, by contrast, commonly employs agents such as KOH, ZnCl_2_, or H_3_PO_4_ to promote dehydration, bond cleavage, and selective burn-off during carbonization, often yielding much higher surface areas and more developed microporosity or hierarchical pore networks [19,20]. In coconut-based systems, KOH-centered routes have been especially effective for generating high surface area and improved capacitive behavior [15,21], while H_3_PO_4_-assisted pretreatment has been used to tune mesoporosity and facilitate ion transport [15]. At the same time, the activation environment itself can alter pore accessibility, defect density, and conductivity, thereby affecting not only capacitance but also rate capability and long-term stability [22]. Thus, activation should be viewed not as a simple surface-area maximization step, but as a structural design tool that determines how efficiently electrolyte ions can reach and utilize the internal carbon surface.

Despite the progress achieved, several important gaps remain in the literature on coconut-fiber-derived activated carbons for supercapacitors. First, many studies still emphasize maximizing Brunauer–Emmett–Teller (BET) surface area, even though increasing surface area alone does not guarantee improved electrochemical performance when pore entrances are constricted or ion transport through the network is sluggish [1,4,6]. Second, the structure–property–performance relationship remains insufficiently resolved: the relative roles of micropore size, mesopore connectivity, defect chemistry, and conductivity are often discussed qualitatively rather than established through systematic design and electrochemical analysis [1,6]. Third, there is a persistent trade-off between creating highly developed porosity and preserving electronic conductivity, since harsh activation can increase disorder and resistance even while enlarging surface area [22,23]. These limitations are especially relevant for coconut-fiber-derived carbons, where precursor variability and processing conditions can lead to markedly different pore architectures and charge-storage behavior.

For these reasons, activated carbon derived from coconut fiber remains a highly relevant model system; however, the field has moved beyond merely demonstrating the feasibility of biomass-to-carbon conversion and now requires a deeper understanding of the relationships between structure, processing, and performance. There is still limited insight into how precursor pre-treatments influence the interaction with activating agents, the evolution of porosity, and ultimately ion transport and electrochemical response. In this context, the present work aims to systematically investigate the influence of different pre-treatment strategies applied to coconut fiber on the properties of activated carbons obtained through KOH chemical activation. Two pre-treatment approaches were evaluated—(i) oxidative calcination at 300 °C in air atmosphere and (ii) hydrothermal treatment at 200 °C for 4 h—together with a reference sample prepared without pre-treatment. We hypothesize that controlled modification of the lignocellulosic structure of the precursor can enhance KOH impregnation and promote the development of a more favorable porous architecture, thereby improving ion accessibility and charge storage behavior. To test this hypothesis, the resulting materials were characterized by XRD, Raman spectroscopy, FTIR, and electrochemical techniques, with the objective of establishing the relationship between precursor conditioning, structural properties, and supercapacitor performance. This study provides insights into the rational design of biomass-derived activated carbons for energy storage applications.

## 2. Materials and Methods

### 2.1. Materials and Chemicals

Coconut fibers were purchased from a company in southeastern Mexico. The size of the fibers was reduced to a length of 0.5 to 1 cm and washed with a 1% *w*/*v* solution of phosphate-free detergent and water; and then, they were dried at 80 °C for 24 h. Potassium hydroxide (KOH, ACS reagent grade, CAS No. 1310-58-3), polyvinylidene fluoride (PVDF, reagent grade, CAS No. 24937-79-9), and dimethylformamide (DMF, 99.9%, CAS No. 68-12-2) were purchased from Sigma-Aldrich (St. Louis, MO, USA) and used as received without further purification.

### 2.2. Synthesis and Chemical Activation of Coconut Fibers

Coconut fibers were used as a lignocellulosic precursor for the synthesis of activated carbons via KOH chemical activation. To elucidate the role of precursor preconditioning on pore development and carbon reactivity, three distinct synthetic routes were systematically investigated. As part of the preconditioning strategy, alkaline treatment was carried out using a NaOH solution at a fixed concentration of 7% (*w*/*w*) for 4 h [24]. Subsequently, the fibers were neutralized with acetic acid, thoroughly washed three times with distilled water to remove residual chemicals, and dried at 80 °C for 24 h to obtain the pretreated material, hereafter denoted as CFs.

In the baseline route, the pretreated coconut fibers (CFs) were directly subjected to chemical activation without further modification. The precursor was impregnated with KOH at a CF:KOH mass ratio of 1:2 using deionized water as dispersing medium. The suspension was maintained under continuous stirring at 80 °C for 24 h to promote solvent evaporation and enhance the diffusion of the activating agent into the carbon matrix. The resulting solid was finely ground to ensure homogeneity and subsequently subjected to a two-step thermal treatment under N_2_ atmosphere: an initial stabilization at 120 °C for 30 min, followed by activation at 700 °C for 1 h using a heating rate of 10 °C min^−1^. After thermal treatment, the system was allowed to cool naturally to room temperature.

To assess the influence of oxidative preconditioning, a fraction of the precursor was subjected to oxidative calcination prior to activation. In this case, CFs were treated at 300 °C for 2 h under air atmosphere, yielding a partially carbonized and oxygen-functionalized intermediate (CF-OC). This pretreated material was ground and subsequently activated under identical conditions to the baseline route. The oxidative step is expected to induce partial devolatilization and incorporation of oxygen-containing functionalities, which can act as reactive sites during KOH activation, potentially modifying pore nucleation and growth dynamics.

In the third route, hydrothermal carbonization was employed as a preconditioning strategy to tailor the precursor structure. CFs were dispersed in deionized water (40 mg mL^−1^) and treated in a sealed reactor at 200 °C for 8 h, producing a hydrochar (CF-HTC). This process promotes dehydration, aromatization, and partial reorganization of the biomass structure, leading to a more condensed carbon framework. After washing, drying (90 °C, 24 h), and grinding, the hydrothermally treated precursor was subjected to the same KOH activation protocol. This route is expected to influence the accessibility of the activating agent and the evolution of the pore network due to the altered physicochemical nature of the hydrochar.

Following thermal activation, all samples were thoroughly washed with H_2_SO_4_ solution and deionized water until neutral pH (6–7) was achieved, ensuring the removal of inorganic residues and potassium-containing byproducts. The materials were then filtered under vacuum and dried at 80 °C overnight.

The resulting activated carbons were denoted as CF-AC (direct activation), CF-OC-AC (oxidative preconditioning), and CF-HTC-AC (hydrothermal preconditioning). The overall yields were ~37%, 31%, and 34%, respectively. The observed variations in yield are indicative of differences in precursor decomposition pathways and carbon retention, which are directly linked to the extent of structural rearrangement induced by each preconditioning strategy.

### 2.3. Characterization of the Activated Carbons

The crystal structure was studied using a Bruker D8 Advance diffractometer (Bruker, Billerica, MA, USA) operating with Cu-Kα radiation (λ = 1.5406 Å) in Bragg–Brentano geometry. XRD patterns were recorded over a 2θ range of 5–60°. Fourier transform infrared (FTIR) spectra were collected using a Bruker Tensor 27 spectrophotometer equipped with a diamond attenuated total reflection (ATR) accessory. Spectra were acquired in the range of 4000–1000 cm^−1^ with a spectral resolution of 4 cm^−1^ and 64 scans per sample. Raman spectra were collected using a Renishaw inVia Raman spectrometer (Renishaw, Wotton-under-Edge, UK) operating in backscattering geometry and equipped with a 633 nm He–Ne laser source. The laser beam was focused on the sample through a 50× objective lens, resulting in a spot size of approximately 1 μm. To minimize laser-induced heating and structural modification of carbon materials, a laser power of 1% was employed during all measurements. Nitrogen adsorption–desorption measurements were carried out in a 3P Micro200 surface area and porosimetry analyzer (3P Instruments, Odelzhausen, Germany). The specific surface area was determined by the Brunauer–Emmett–Teller (BET) method from the N_2_ adsorption–desorption isotherms, while the average pore diameter was obtained from the corresponding porosimetry analysis.

### 2.4. Electrochemical Tests

The electrochemical performance of the synthesized activated carbon materials was first evaluated in a three-electrode configuration. The working electrodes were prepared by dispersing porous carbon, conductive carbon black, and polytetrafluoroethylene (PTFE) in a mass ratio of 8:1:1, followed by drying at 80 °C. A small amount of ethanol was then added to form a homogeneous slurry, which was coated onto pre-treated glassy carbon substrates. The electrodes were subsequently immersed in 1 M H_2_SO_4_ overnight to ensure proper electrolyte infiltration.

Electrochemical measurements were carried out using a CS350M electrochemical workstation (Wuhan CorrTest Instrument Co., Ltd., Wuhan, China). A conventional three-electrode system was employed, consisting of a platinum counter electrode and an Ag/AgCl reference electrode in 1 M H_2_SO_4_ electrolyte. Cyclic voltammetry (CV) was performed within a potential window of −0.2 to 0.8 V at scan rates ranging from 1 to 50 mV s^−1^. Galvanostatic charge–discharge (GCD) tests were conducted at current densities between 0.5 and 5 A g^−1^.

The specific capacitance of the working electrodes was first estimated from the CV curves using Equation (1):

(1) $$C_{spc}=\frac{1}{2mv\Delta V}\int IdV,$$

In addition, the capacitance was independently evaluated from the galvanostatic charge–discharge (GCD) profiles according to:

(2) $$C_{G}=\frac{I\Delta t}{m\Delta V}\;\;,$$

where *I* is the discharge current (A), Δ*t* is the discharge time (s), *m* is the mass of active material (g), and Δ*V* is the potential window (V).

## 3. Results

### 3.1. Structural and Textural Properties

**Figure 1:**
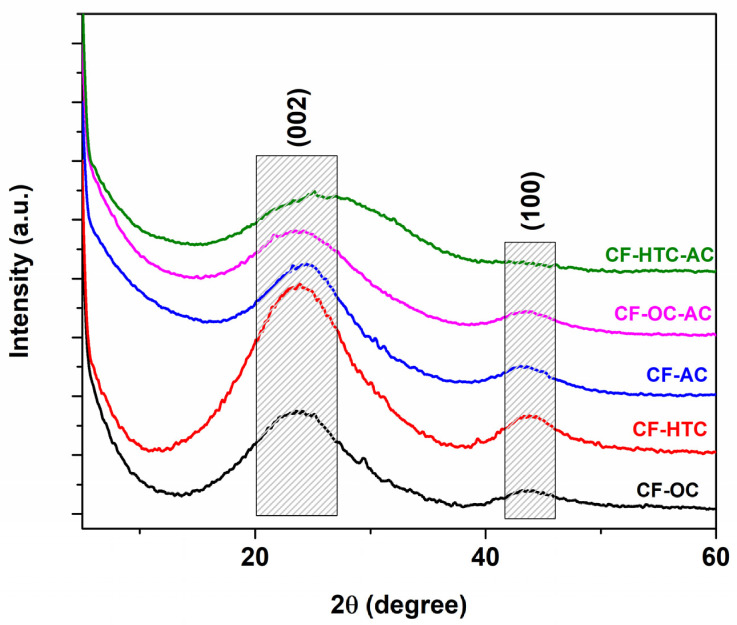
XRD patterns of the carbon samples derived from coconut fiber, showing the characteristic (002) and (100) reflections of turbostratic carbon structures.

The X-ray diffraction (XRD) patterns of all samples (Figure 1) exhibit two broad diffraction features centered in the ranges of 22–27° and 42–45°, corresponding to the (002) and (100) planes of carbon materials, respectively. The broadness and low intensity of these reflections indicate that all samples possess a predominantly amorphous/turbostratic carbon structure with limited long-range order, as commonly observed in biomass-derived activated carbons [25,26]. A more detailed structural analysis was performed from the (002) reflection (Table 1), allowing the estimation of the interlayer spacing (d002), crystallite height (Lc), and the number of stacked graphene layers (N). The calculated d002 values range from 0.336 to 0.374 nm, which are larger than that of ideal graphite (~0.335 nm), confirming the presence of expanded and disordered carbon layers. This expansion is typically associated with defect formation and structural rearrangements induced during chemical activation, which disrupt graphitic stacking and reduce crystallite size [27,28].

The pretreated samples, CF-OC and CF-HTC, exhibit similar structural parameters, with d002 values of 0.374 and 0.372 nm and Lc values close to 0.80 nm, corresponding to approximately three stacked graphene layers. This indicates that both oxidative and hydrothermal pretreatments lead to carbon structures with comparable short-range ordering and a moderate degree of structural disorder, as commonly reported for partially carbonized biomass precursors [29]. However, significant differences arise after KOH activation depending on the pretreatment route. The CF-AC sample shows a reduction in d002 (0.360 nm), along with an increase in Lc (1.05 nm) and the number of stacked layers (~3.9), suggesting a slight increase in structural ordering, likely associated with the rearrangement of carbon domains during activation. In contrast, CF-OC-AC maintains a relatively disordered structure, with intermediate values of d002 (0.371 nm) and Lc (0.83 nm). This behavior suggests that, under the specific conditions employed in this study, oxidative pretreatment does not significantly enhance structural reorganization during activation, although such effects have been reported to depend strongly on oxidation severity and activation parameters in biomass-derived carbons [30].

A markedly different behavior is observed for CF-HTC-AC, which exhibits the broadest (002) peak (FWHM = 12.7°), the lowest L_c_ value (0.64 nm), and the smallest number of stacked layers (~2.9), along with a shift in the (002) reflection toward higher diffraction angles (26.5°). This combination of features indicates a strong disruption of graphitic stacking and the formation of highly defective carbon domains with reduced crystallite size. Additionally, the absence of a clearly distinguishable (100) reflection suggests a loss of in-plane structural coherence, further confirming the highly disordered nature of this sample.

To further elucidate the structural disorder and short-range ordering suggested by the XRD analysis, Raman spectroscopy was employed as a complementary technique highly sensitive to the local bonding configuration and defect density of carbon materials. While XRD provides information on long-range ordering and stacking of graphene layers, Raman spectroscopy enables a more detailed assessment of the degree of disorder, graphitic domain size, and the nature of defects within the carbon framework.

**Figure 2:**
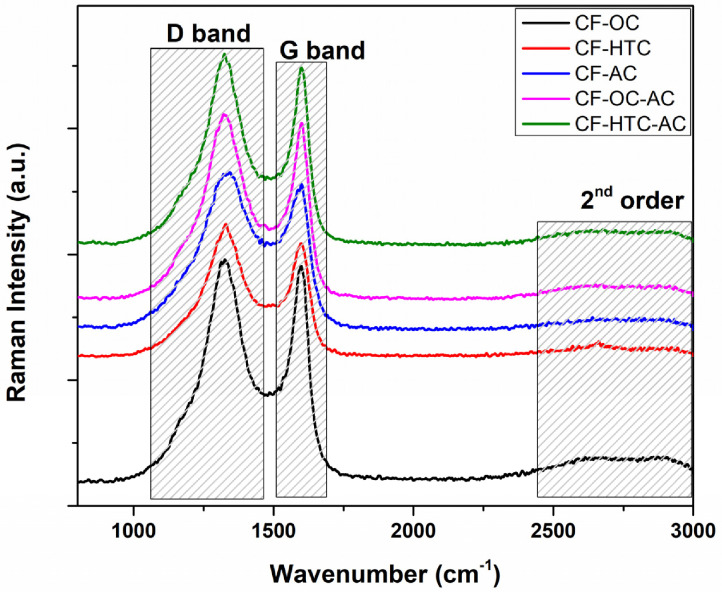
Raman spectra of the carbon samples derived from coconut fiber.

The Raman spectra of all samples (Figure 2) exhibit the characteristic D (~1350 cm^−1^) and G (~1590 cm^−1^) bands, which are commonly observed in disordered carbon materials. The G band is associated with the in-plane vibration of sp^2^-hybridized carbon atoms and originates from the Raman-allowed E_2g_ phonon mode of graphite, whereas the D band arises from a defect-induced double-resonance Raman scattering process and is therefore activated only in the presence of structural disorder and finite crystallite size [31]. This confirms that all samples possess a turbostratic carbon structure, in agreement with the XRD results.

**Figure 3:**
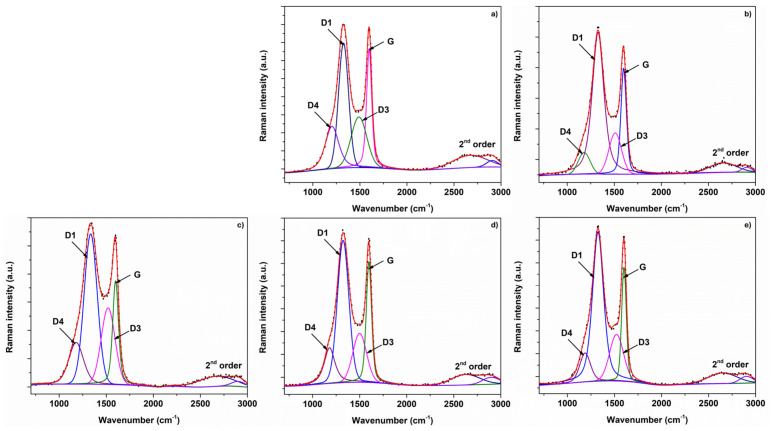
Deconvoluted Raman spectra of the carbon samples derived from coconut fiber: (**a**) CF-OC, (**b**) CF-HTC, (**c**) CF-AC, (**d**) CF-OC-AC, and (**e**) CF-HTC-AC.

A more detailed analysis was performed through spectral deconvolution (Figure 3). The D3 band and the second-order Raman bands were fitted using Gaussian functions, whereas the D1, D2, D4, and G bands were fitted using pseudo-Voigt functions. This approach allowed the identification of additional bands such as D3 (~1500 cm^−1^), related to amorphous carbon, and D4 (~1200 cm^−1^), typically associated with sp^3^ carbon or functional groups [32]. The presence and relative intensity of these bands further support the coexistence of disordered and partially graphitic domains within the carbon structure.

The ID/IG ratio was used as an indicator of structural disorder (Table 2). An increase in ID/IG was observed after KOH activation, with CF-AC exhibiting the highest value, indicating a significant reduction in graphitic domain size and an increase in defect density. However, as widely reported, this parameter should not be interpreted solely as a direct measure of graphitization, since it is also influenced by the crystallite size and the excitation conditions, as described by [31,33]. Therefore, complementary parameters were considered for a more comprehensive interpretation.

In this regard, the AD1/AG ratio provided a more sensitive evaluation of the structural evolution. A notable increase in this parameter was observed for the hydrothermally treated samples, particularly CF-HTC-AC, suggesting that the HTC process promotes the formation of chemically active defect sites in addition to structural disorder [34]. This behavior indicates that hydrothermal treatment contributes to the incorporation of functional groups and heteroatom-related defects, which are not fully captured by ID/IG alone.

Further insight was obtained from the analysis of the full width at half maximum (FWHM) of the G band. Although CF-AC exhibited the highest ID/IG ratio, it also presented the broadest G band, indicating a highly amorphous structure rather than well-defined defective graphitic domains [35,36]. In contrast, CF-HTC-AC displayed a relatively narrower G band combined with a high defect density, suggesting a more favorable balance between structural disorder and electronic conductivity.

Additionally, the broad and low intensity 2D band observed in all samples confirms the absence of long-range graphitic ordering and the predominance of turbostratic carbon structures, consistent with the XRD results. Similar features have been reported in activated carbons derived from biomass, where excessive activation leads to the disruption of graphitic stacking and the formation of highly defective carbon frameworks [37].

**Figure 4:**
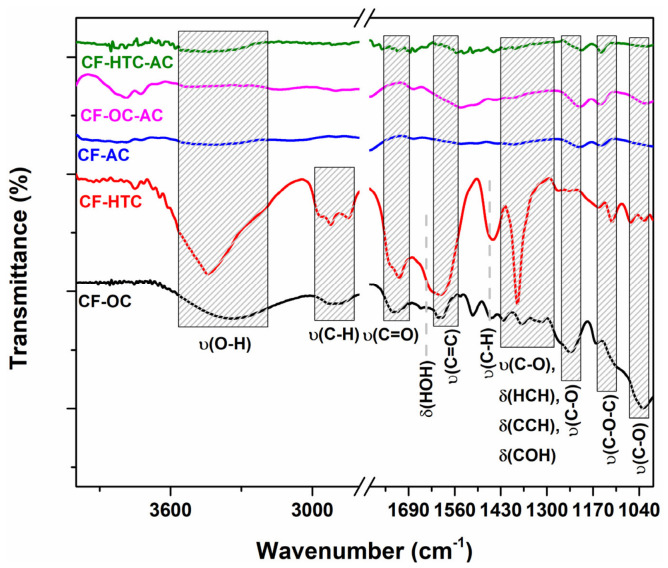
FTIR spectra of coconut-fiber-derived samples before and after KOH activation.

The surface chemistry of the coconut-fiber-derived carbons and its evolution after different pre-treatment strategies and subsequent activation were analyzed by FTIR spectroscopy (Figure 4).

The spectra of the pre-treated samples exhibit several well-defined absorption bands, indicating the presence of oxygen-containing functional groups generated or preserved during the pre-treatment stage. A broad band centered around ~3400 cm^−1^ is observed in both CF-OC and CF-HTC samples, corresponding to ν(O–H) stretching vibrations associated with hydroxyl groups and/or adsorbed moisture [38]. This band is notably wide in both cases, reflecting a distribution of hydrogen-bonded species; however, it is less intense in the oxidatively treated sample (CF-OC) compared to CF-HTC, suggesting a relatively lower abundance of hydroxyl and polar oxygenated functionalities. Although this feature is still detectable in the activated materials, its intensity is significantly reduced, indicating the progressive removal of O–H-containing groups during thermal annealing and KOH activation.

In the region between 1700 and 1730 cm^−1^, a distinct band is detected, typically assigned to ν(C = O) stretching vibrations from carbonyl and carboxylic groups [39,40]. The presence and intensity of this band indicate that both oxidative and hydrothermal pre-treatments promote the incorporation or preservation of oxygenated functionalities within the lignocellulosic structure. Additionally, a band around ~1550–1620 cm^−1^ is observed, commonly associated with ν(C = C) skeletal vibrations of aromatic domains [41,42]. However, contributions from the bending vibration of adsorbed water, δ(HOH), near 1640 cm^−1^ cannot be excluded, particularly for the CF-OC and CF-HTC samples, which also exhibit a pronounced ν(O–H) band in the 3200–3600 cm^−1^ region [43].

For the CF-HTC and CF-OC samples, absorption bands in the 2850–2950 cm^−1^ region are attributed to ν(C–H) stretching vibrations of methyl and methylene groups, while the band near 1450 cm^−1^ is associated with δ(HCH) bending vibrations [44,45]. These features suggest that part of the aliphatic structure of the biomass is preserved after hydrothermal treatment.

Further contributions are detected in the range of 1000–1430 cm^−1^, corresponding to ν(C–O) and ν(C–O–C) stretching modes, as well as δ(HCH), δ(CCH), and δ(COH) bending vibrations, which are typically associated with alcohol, ether, epoxide, and other oxygen-containing functional groups [38,45]. The presence and relative intensity of these bands indicate that the oxidative and hydrothermal pre-treatments significantly modify the surface chemistry of the precursor, promoting the incorporation and preservation of oxygenated functionalities that may influence both the interaction with the activating agent and the electrochemical behavior of the resulting carbons.

**Figure 5:**
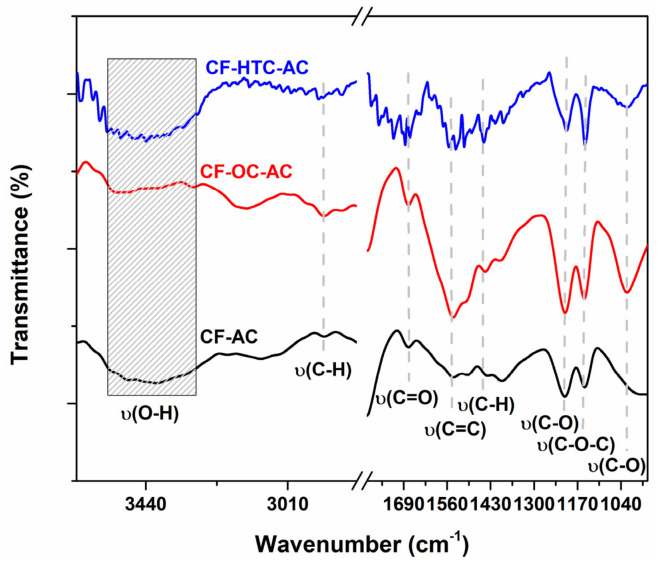
FTIR spectra of KOH-activated coconut-fiber-derived carbons.

After the thermal annealing and subsequent KOH activation, a drastic reduction in the intensity of most FTIR bands is observed (Figure 5). In particular, the signals associated with O–H and C = O groups become significantly weaker or nearly disappear, indicating the progressive decomposition of oxygenated functionalities at high temperatures. This behavior is consistent with the thermal instability of labile surface groups and the formation of a more carbonized structure during activation [46]. The fine spectral features observed in the CF-HTC-AC spectrum are likely associated, at least in part, with residual atmospheric water vapor contributions (vibrational–rotational water vapor artifacts), which are commonly observed in FTIR measurements and do not affect the overall interpretation of the spectra.

The attenuation or disappearance of these bands suggests that the activation process leads to partial deoxygenation of the carbon matrix, favoring the development of a more condensed aromatic structure. This transformation agrees with the XRD results, where broad diffraction peaks associated with turbostratic carbon are observed, indicating a low degree of crystallinity but increased structural ordering after activation. Similarly, Raman spectroscopy reveals the dominance of the D and G bands, characteristic of disordered graphitic structures, further supporting the transition from a functionalized precursor to a defective carbon framework.

It is important to note that the interpretation of FTIR spectra in carbon-based materials must be approached with caution, as band assignments can be affected by overlapping vibrational modes and the presence of adsorbed species. Furthermore, since the spectra were acquired using the ATR-FTIR technique, the obtained information is primarily associated with the near-surface region of the material due to the limited penetration depth of the evanescent wave, which typically extends only a few micrometers into the sample. Therefore, the identification of functional groups is based on commonly accepted assignments in literature and should be considered in conjunction with complementary techniques such as XRD and Raman spectroscopy.

**Figure 6:**
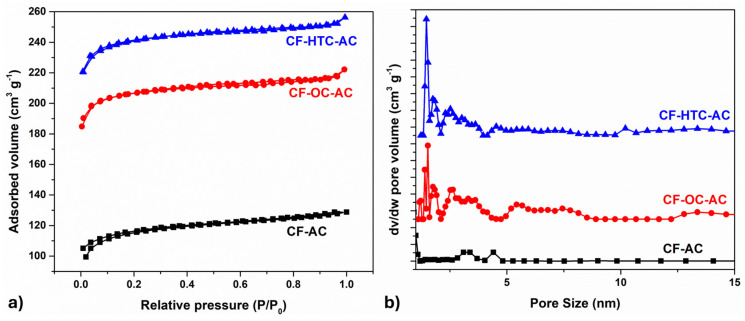
(**a**) N_2_ adsorption–desorption isotherms and (**b**) pore size distributions for CF-AC, CF-OC-AC, and CF-HTC-AC activated carbons.

To evaluate the effect of the different pre-treatment strategies on pore development, N_2_ adsorption–desorption measurements were performed. The resulting textural parameters are summarized in Table 3, while the adsorption–desorption isotherms and pore size distributions are shown in Figure 6.

The directly activated sample (CF-AC) exhibited a BET surface area of 460.1 m^2^ g^−1^ and a total pore volume of 0.24 cm^3^ g^−1^. In contrast, both pre-treated samples showed substantially enhanced textural properties after KOH activation. The BET surface area increased to 832.4 m^2^ g^−1^ for CF-OC-AC and reached 975.2 m^2^ g^−1^ for CF-HTC-AC, while the total pore volume increased to 0.29 and 0.30 cm^3^ g^−1^, respectively. These results indicate that both oxidative and hydrothermal pre-treatments promote a more efficient activation process, with the hydrothermal route producing the most developed porous structure.

The adsorption–desorption isotherms shown in Figure 6a reveal a pronounced uptake at low relative pressures for all samples, indicating the development of a predominantly microporous structure during KOH activation. The isotherms exhibit characteristics commonly associated with Type I behavior, which is typical of microporous activated carbons [47], although a gradual increase in adsorption at higher relative pressures suggests the presence of larger pores contributing to the overall pore volume. However, the amount of adsorbed nitrogen follows the order CF-HTC-AC > CF-OC-AC > CF-AC, in agreement with the measured BET surface areas. The higher adsorption capacity of CF-HTC-AC throughout the entire pressure range suggests the formation of a more extensive and accessible pore network, which is expected to facilitate electrolyte transport and enhance the utilization of electrochemically active surface sites [47].

The pore size distributions presented in Figure 6b provide additional insight into the effect of precursor conditioning on pore development. CF-AC displays a limited pore volume mainly concentrated in the micropore region, whereas both pre-treated samples exhibit broader pore size distributions and higher pore volumes. CF-HTC-AC exhibits a broader pore size distribution with contributions extending toward the mesopore region. Such pore characteristics are generally considered favorable for supercapacitor electrodes because micropores provide abundant surface area for charge storage, whereas larger pores improve electrolyte accessibility and ion transport within the carbon framework [48,49,50]. The combination of high surface area and improved pore accessibility is expected to facilitate electrolyte transport and increase the utilization of electrochemically active sites, thereby contributing to the enhanced capacitance and rate capability of pre-treated carbons [48,49,50].

The marked increase in surface area and pore volume can be related to the chemical changes induced by the pre-treatment routes. As discussed in the FTIR analysis, oxidative and hydrothermal treatments promote the formation or preservation of oxygen-containing functional groups, particularly hydroxyl and carbonyl species. These functionalities may act as preferential anchoring sites for potassium-containing species during KOH impregnation, promoting a more homogeneous activation process and enhancing pore generation during thermal treatment [49]. This interpretation is consistent with the substantially higher BET surface areas obtained for the pre-treated samples.

Beyond surface area, pore width also plays an important role in ion accessibility. CF-HTC-AC exhibited the largest average pore width (1.51 nm) compared with 1.20 nm for CF-AC and 1.15 nm for CF-OC-AC. Although all samples are predominantly microporous, the slightly wider pores developed in CF-HTC-AC are expected to facilitate electrolyte ion transport and improve the utilization of the internal surface area [48,50]. Therefore, the superior capacitance and rate capability observed for CF-HTC-AC cannot be attributed solely to its higher BET surface area, but rather to the combined effect of increased surface area, larger pore volume, and a more favorable pore architecture for charge storage and ion transport [48]. This observation is consistent with the electrochemical results presented in the following section, where CF-HTC-AC exhibits the highest specific capacitance and superior rate capability among the investigated samples.

### 3.2. Electrochemical Performance of the Electrode

**Figure 7:**
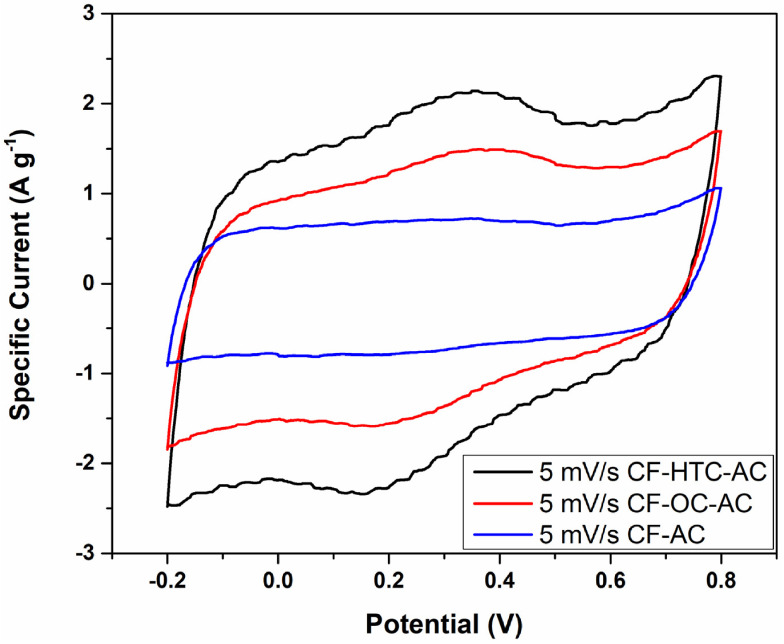
Cyclic voltammetry (CV) curves of CF-AC, CF-OC-AC, and CF-HTC-AC electrodes measured at a scan rate of 5 mV s^−1^ in 1 M H_2_SO_4_ using a three-electrode configuration.

Figure 7 compares the CV profiles of CF-AC, CF-OC-AC, and CF-HTC-AC measured at 5 mV s^−1^ in 1 M H_2_SO_4_. All electrodes exhibit capacitive-type responses, although the pretreated samples show a broader enclosed area and slight deviations from an ideal rectangular shape. CF-OC-AC and CF-HTC-AC display weak redox humps, suggesting the contribution of surface faradaic reactions in addition to electric double-layer capacitance. These features can be attributed to the presence of oxygen-containing functional groups introduced or preserved during the oxidative and hydrothermal pretreatments [51,52], as confirmed by FTIR analysis (Figure 5), including carbonyl (C = O), hydroxyl (–OH), and C–O functionalities. In acidic aqueous electrolytes, such oxygenated groups are known to participate in reversible or quasi-reversible proton-coupled surface reactions, contributing additional pseudocapacitance to carbon-based electrodes [53,54,55].

The stronger current response observed for CF-HTC-AC indicates a higher charge-storage capability compared with CF-AC and CF-OC-AC. This behavior suggests that hydrothermal pretreatment favored a more electrochemically accessible surface through a combination of oxygenated surface chemistry and improved pore accessibility.

The slight fluctuations and deviations from the ideal rectangular shape observed in the CV profiles, particularly for the CF-HTC-AC electrode, can be attributed to the intrinsic characteristics of highly porous carbon materials rather than to parasitic electrochemical processes. Previous studies have demonstrated that charge storage in porous carbons does not occur uniformly throughout the electrode structure. Instead, ion transport and charge accumulation are distributed across pore networks with different sizes, tortuosities, and accessibility, resulting in a broad distribution of relaxation times and localized differences in charging kinetics. Consequently, the electrochemical response may exhibit minor distortions or current fluctuations even when the overall behavior remains predominantly capacitive. These effects become more pronounced in materials with hierarchical porosity, where electrolyte ions simultaneously access external surfaces, mesopores, and deeper microporous regions through pathways characterized by different diffusion rates [56,57].

Furthermore, the electrochemically accessible pore volume has been identified as a critical parameter influencing the shape and scan-rate dependence of cyclic voltammograms in porous carbon electrodes. The contribution of charge storage from internal pore domains may not be perfectly synchronized with that of the external surface, leading to transient charge redistribution phenomena and small departures from an ideal electric double-layer response. Similar behavior has been reported for porous carbon electrodes where distributed capacitance, pore connectivity, and diffusion limitations generate subtle irregularities in the current response without compromising electrochemical reversibility or capacitive performance. Therefore, the minor fluctuations observed for CF-HTC-AC are consistent with the enhanced pore accessibility and hierarchical porous structure promoted by the hydrothermal pretreatment and subsequent KOH activation [56,57,58].

To further evaluate electrochemical performance, the specific capacitance as a function of scan rate was analyzed and is presented in Figure 7. A clear dependence of capacitance on scan rate is observed for all samples, where the capacitance decreases with increasing scan rate. This behavior is commonly attributed to limitations in ion diffusion within the porous structure, as electrolyte ions are unable to fully access micropores at higher scan rates [59,60].

**Figure 8:**
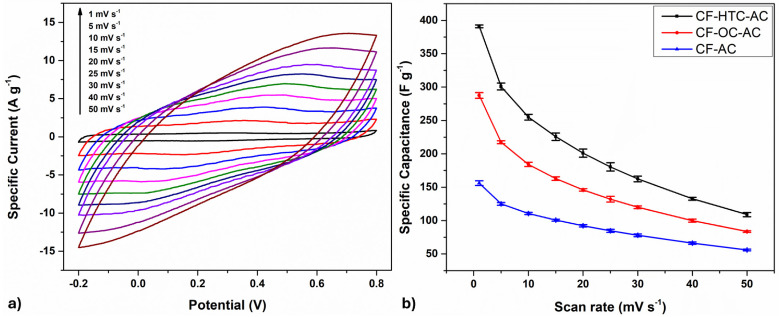
(**a**) CV curves of the CF-HTC-AC electrode measured at different scan rates, and (**b**) specific capacitances of CF-AC, CF-OC-AC, and CF-HTC-AC electrodes as a function of scan rate in 1 M H_2_SO_4_ using a three-electrode configuration.

Among the evaluated materials, CF-HTC-AC exhibits the highest specific capacitance across the entire range of scan rates, as well as improved capacitance retention at higher scan rates. This behavior suggests a more efficient ion transport and a more accessible pore network, which can be attributed to hydrothermal pretreatment. In contrast, CF-AC shows a more pronounced decay in capacitance with increasing scan rate, indicating restricted ion diffusion and limited utilization of the available surface area. The superior performance of CF-HTC-AC is further supported by the shape of the CV curves at different scan rates (Figure 8b), where the electrode maintains a relatively stable profile even at higher scan rates. This indicates good rate capability and low internal resistance, which are essential characteristics for high-performance supercapacitor electrodes.

To place the electrochemical performance of the prepared materials in context, a comparison with previously reported biomass-derived activated carbons, including systems prepared through hydrothermal and oxidative pretreatments, is presented in Table 4. As shown, various lignocellulosic precursors such as pineapple leaf fiber, oyster mushrooms, sugarcane bagasse, and coconut husk have been successfully converted into activated carbons exhibiting specific capacitances in the range of ~130 to 370 F g^−1^ under different electrolytes and testing conditions. Notably, the CF-HTC-AC sample developed in this work delivers a specific capacitance of ~332 F g^−1^ at 0.5 A g^−1^ in 1 M H_2_SO_4_, which is comparable to or higher than many previously reported systems. This performance highlights the effectiveness of the combined hydrothermal pretreatment and KOH activation strategy in enhancing the electrochemical properties of biomass-derived carbons. Furthermore, the results demonstrate that coconut fiber, as a low-cost and abundant precursor, can be effectively engineered to achieve competitive energy storage performance relative to other biomass sources reported in the literature.

A comparison with recent biomass-derived activated carbons reveals that pre-treatment strategies play a decisive role in defining the balance between surface functionality, pore development, and electrochemical performance. Qin et al. reported that hydrothermal pre-treatment of coconut fiber prior to KOH activation produced activated carbons with specific capacitances up to 315.5 F g^−1^ [15]. Their results demonstrated that hydrothermal conditioning promotes the formation of a hierarchical pore structure with improved ion accessibility, while simultaneously preserving oxygen-containing functionalities that contribute to charge storage. Similar behavior is observed in the present work, where the hydrothermally pre-treated sample (CF-HTC-AC) exhibited the highest BET surface area, pore volume, and capacitance among the evaluated materials.

Interestingly, the oxidative pre-treatment route employed in this study also proved highly effective. A comparable strategy was previously reported by Kayode et al. for brewery bagasse-derived activated carbons, where oxidative calcination prior to KOH activation enhanced pore development and electrochemical performance [66]. However, the capacitance reported for the oxidatively treated brewery bagasse (196.15 F g^−1^) remained considerably lower than that obtained for the oxidatively treated coconut fiber in the present work (332.1 F g^−1^). This difference suggests that, beyond the activation route itself, the intrinsic composition and structural characteristics of the biomass precursor strongly influence the final pore architecture and electrochemical response.

To gain deeper insight into the charge storage mechanism, the electrochemical kinetics of the electrodes were further analyzed using both power-law analysis and Dunn’s method. The relationship between current response (*i*) and scan rate (*v*) can be described by [67]:

(3) $$i={av}^{b},$$

(4) $$\log\left(i\right)=\left(a\right)+b\times log(v),$$

where the *b*-value provides insight into the dominant charge storage mechanism. A *b*-value approaching 1 indicates a surface-controlled capacitive process (electric double-layer capacitance, EDLC), whereas a value close to 0.5 suggests a diffusion-controlled process associated with faradaic reactions. In carbon-based electrodes, intermediate *b*-values typically reflect a mixed charge storage mechanism involving both EDLC and pseudocapacitance, as is widely reported in the literature [68,69].

To quantitatively evaluate these contributions, Dunn’s method was applied according to:

(5) $$i\left(V\right)=k_{1}v+k_{2}v^{1/2}$$

where *k*_1_*v* represents the surface-controlled (capacitive) contribution and *k*_2_*v*^1/2^ corresponds to the diffusion-controlled contribution. This approach enables the separation of the total current into capacitive and diffusion-controlled components at different scan rates.

**Figure 9:**
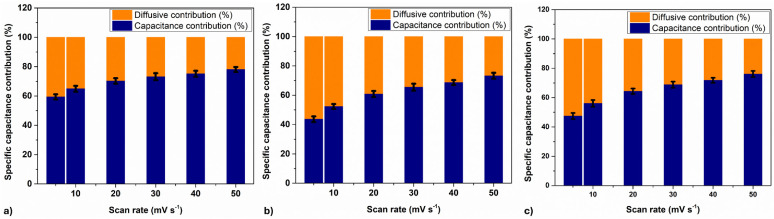
Separation of capacitive and diffusion-controlled contributions based on Dunn’s method for (**a**) CF-AC, (**b**) CF-OC-AC, and (**c**) CF-HTC-AC electrodes at different scan rates in 1 M H_2_SO_4_ using a three-electrode configuration.

Figure 9 presents the relative contributions of capacitive and diffusion-controlled processes for CF-AC, CF-OC-AC, and CF-HTC-AC electrodes. For all samples, the diffusion-controlled contribution dominates at low scan rates, indicating that electrolyte ions have sufficient time to penetrate the porous structure and interact with electrochemically active sites. As the scan rate increases, the capacitive contribution progressively increases, reflecting the transition toward surface-controlled processes due to limited ion diffusion, as is commonly observed in porous carbon electrodes [70].

Among the evaluated materials, CF-OC-AC exhibits the highest diffusion-controlled contribution across the entire range of scan rates (from ~56% at 5 mV s^−1^ to ~24% at 50 mV s^−1^), indicating a significant involvement of faradaic processes. This behavior is consistent with the presence of oxygen-containing functional groups identified by FTIR, which can participate in reversible proton-coupled redox reactions in acidic media, contributing to pseudocapacitance. The CF-HTC-AC sample shows a very similar but slightly lower diffusion-controlled contribution (from ~52% to ~25%), suggesting that hydrothermal pretreatment also promotes the formation of electrochemically active surface functionalities and enhances ion accessibility. Despite this marginal difference in diffusion contribution, CF-HTC-AC delivers superior overall electrochemical performance, indicating a more favorable balance between capacitive and diffusion-controlled processes. In contrast, CF-AC presents the lowest diffusion-controlled contribution at higher scan rates (down to ~21%), indicating a more surface-limited charge storage mechanism dominated by electric double-layer capacitance (EDLC).

These results highlight that maximizing pseudocapacitive contributions alone does not necessarily lead to optimal electrochemical performance. Although CF-OC-AC exhibits the highest diffusion-controlled contribution, CF-HTC-AC delivers superior capacitance and rate capability. This behavior suggests that the overall performance is governed by the synergistic combination of accessible surface area, pore volume, pore size distribution, and surface functionality, which together promote efficient ion transport while maintaining significant charge-storage activity.

**Figure 10:**
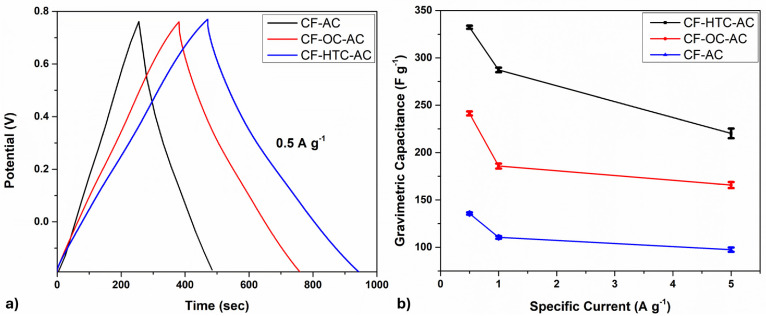
(**a**) Galvanostatic charge–discharge (GCD) curves of CF-AC, CF-OC-AC, and CF-HTC-AC electrodes at a current density of 0.5 A g^−1^, and (**b**) gravimetric capacitance as a function of current density for the same electrodes in 1 M H_2_SO_4_ using a three-electrode configuration.

Additional insight into the charge-storage behavior was obtained through galvanostatic charge–discharge (GCD) measurements at different current densities (Figure 10). All electrodes exhibit nearly symmetric charge–discharge profiles, indicating good electrochemical reversibility. However, slight deviations from ideal linearity are observed, particularly for the pretreated samples, suggesting the coexistence of electric double-layer capacitance and pseudocapacitive contributions, in agreement with the CV and Dunn’s analysis.

At a current density of 0.5 A g^−1^, CF-HTC-AC shows the longest discharge time, confirming its superior charge storage capability, followed by CF-OC-AC and CF-AC. The corresponding gravimetric capacitance values decrease with increasing current density for all samples, which is attributed to limited ion diffusion and reduced accessibility of micropores at higher current densities, as commonly reported for porous carbon electrodes [26]. Notably, CF-HTC-AC retains a higher capacitance across the entire current range, demonstrating improved rate capability. This enhanced performance suggests a more favorable balance between capacitive and diffusion-controlled processes, where efficient ion transport and optimized pore accessibility play a dominant role. In contrast, CF-AC exhibits lower capacitance and a more pronounced drop at higher current densities, indicating a predominantly surface-limited charge storage mechanism.

Although CF-OC-AC exhibits a slightly higher diffusion-controlled contribution, as revealed by Dunn’s analysis, its overall electrochemical performance remains inferior to that of CF-HTC-AC. This result indicates that a higher pseudocapacitive contribution does not necessarily translate into superior rate capability, emphasizing the importance of achieving an optimal balance between pore structure, ion transport, and surface functionality.

**Figure 11:**
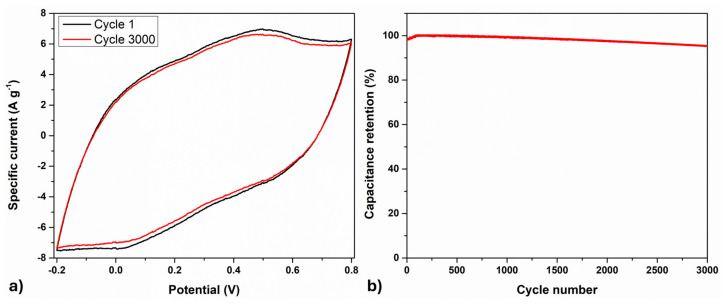
(**a**) Cyclic voltammograms of the CF-HTC-AC electrode recorded during the first and 3000th cycles at a scan rate of 20 mV s^−1^ and (**b**) capacitance retention as a function of cycle number during 3000 continuous CV cycles.

Finally, the cycling stability of the best-performing electrode (CF-HTC-AC) was further evaluated by continuous cyclic voltammetry measurements at a scan rate of 20 mV s^−1^ for 3000 cycles (Figure 11). As shown in Figure 11a, the cyclic voltammograms recorded after the first and the 3000th cycle retain a very similar shape, indicating good electrochemical reversibility and stable charge-storage behavior during long-term operation. Only a slight decrease in current response is observed after 3000 continuous cycles.

Recent studies have highlighted that precise control of structural features can significantly improve electrochemical stability and charge-storage behavior by enhancing the accessibility and reversibility of electrochemically active sites while mitigating degradation during repeated cycling [71]. The capacitance retention during the cycling test is presented in Figure 11b. An initial activation process is observed during the first cycles, followed by a highly stable electrochemical response. The specific capacitance decreased from 201.1 F g^−1^ in the first cycle to 191.5 F g^−1^ after 3000 cycles, corresponding to a capacitance retention of 95.2%. Such retention is comparable to or higher than many biomass-derived activated carbons reported in the literature, highlighting the robustness of the CF-HTC-AC electrode under prolonged electrochemical operation. These results further support the suitability of this material for supercapacitor applications.

## 4. Conclusions

This work demonstrates that the electrochemical performance of coconut-fiber-derived activated carbons is strongly influenced by the nature of the precursor pre-treatment applied prior to chemical activation. Although all samples were produced under identical KOH activation conditions, the initial modification of the lignocellulosic structure significantly affected the evolution of the carbon framework, resulting in distinct structural, chemical, and electrochemical properties.

Among the evaluated strategies, hydrothermal pre-treatment proved to be the most effective route for enhancing charge-storage performance. XRD and Raman analyses revealed that CF-HTC-AC developed a more disordered carbon structure with reduced crystallite stacking and increased structural heterogeneity, while FTIR results indicated the preservation of surface functionalities that may contribute to improved electrode–electrolyte interactions. These structural characteristics translated into the highest specific capacitance and superior rate capability among the investigated materials, suggesting improved accessibility of electrochemically active sites and more efficient ion transport pathways.

In contrast, oxidative pre-treatment promoted a larger contribution of diffusion-controlled charge-storage processes, as evidenced by Dunn’s kinetic analysis. This behavior indicates a stronger participation of surface redox reactions associated with oxygen-containing functionalities. However, the increased pseudocapacitive contribution did not result in superior electrochemical performance, highlighting that charge-storage efficiency depends not only on surface chemistry but also on the accessibility and connectivity of the carbon structure.

The results further demonstrate that the electrochemical behavior of biomass-derived carbons cannot be explained solely by traditional descriptors such as defect density or degree of graphitic ordering. Instead, performance emerges from the combined influence of structural disorder, surface chemistry, pore accessibility, and charge-storage kinetics. The hydrothermal route appears particularly effective in balancing these factors, leading to enhanced capacitive behavior.

Overall, this study identifies precursor pre-conditioning as a critical design parameter for tailoring activated carbons derived from lignocellulosic biomass. The hydrothermal strategy provides a simple, scalable, and sustainable approach for improving electrode performance and offers valuable insight into the rational development of biomass-based carbon materials for next-generation supercapacitor applications.

Future work should explore the influence of hydrothermal processing parameters on pore development and evaluate the performance of the resulting carbons in practical two-electrode configurations to further assess their potential for scalable energy-storage applications.

## Figures and Tables

**Table 1:** Structural parameters derived from XRD analysis of the carbon samples.

Sample	2θ (002)	FWHM (002)	d_002_ (nm)	L_c_	N Layers	2θ (100)
CF-OC	23.8°	10.0	0.374	0.80	3.1	43.9
CF-HTC	23.9°	10.1	0.372	0.80	3.1	44.0
CF-AC	24.7°	7.7	0.360	1.05	3.9	43.7
CF-OC-AC	23.9°	9.7	0.371	0.83	3.2	43.7
CF-HTC-AC	26.5°	12.7	0.336	0.64	2.9	Not observed

**Table 2:** Structural parameters derived from Raman spectroscopy analysis of the carbon samples.

Sample	D1 (cm^−1^)	G (cm^−1^)	FWHM (G)	ID/IG	AD1/AG
CF-OC	1326.0 ± 0.3	1598.4 ± 0.1	64.0 ± 0.4	1.05	1.67
CF-HTC	1327.8 ± 0.2	1599.4 ± 0.1	68.8 ± 0.2	1.34	3.07
CF-AC	1334.6 ± 0.3	1599.1 ± 0.1	72.6 ± 0.4	1.45	2.72
CF-OC-AC	1324.3 ± 0.4	1598.4 ± 0.1	66.5 ± 0.7	1.18	2.24
CF-HTC-AC	1325.3 ± 0.2	1601.3 ± 0.1	58.2 ± 0.4	1.31	3.42

**Table 3:** Textural properties of CF-AC, CF-OC-AC, and CF-HTC-AC activated carbons. Values are calculated from the adsorption–desorption isotherms of N_2_.

Sample	BET Surface Area (m^2^ g^−1^)	Pore Volume (cm^3^ g^−1^)	Pore Width (nm)
CF-AC	460.1	0.24	1.20
CF-OC-AC	832.4	0.29	1.15
CF-HTC-AC	975.2	0.30	1.51

**Table 4:** Comparison of the properties of porous carbon materials in three electrode systems.

Biomass	Pretreatment	Activator	Electrolyte	Current Density (A g^−1^)/Scan Rate (mV s^−1^)	Specific Capacitance (F g^−1^)	Reference
Pineapple leaf fiber	H_2_SO_4_-assisted HTC	KOH	1 M Na_2_SO_4_	5 mV s^−1^	131.3	[61]
Oyster mushrooms	Precarbonization	KOH	6 M KOH	1 A g^−1^	318.6	[62]
Sugarcane bagasse	Precarbonization	KOH	6 M KOH	0.5 A g^−1^	370	[63]
Coconut husk	Precarbonization	ZnCl_2_	1 M H_2_SO_4_	1 A g^−1^	236	[16]
Camellia seed shell	Precarbonization	KOH	3 M KOH	0.5 A g^−1^	335.9	[64]
Coconut shell	Precarbonization	KOH	6 M KOH	1 A g^−1^	260	[21]
Coconut shell	Hydrothermal treatment	ZnCl_2_	0.5 M H_2_SO_4_	0.25 A g^−1^	250	[65]
Brewery bagasse	Oxidative calcination	KOH	1 M H_2_SO_4_	0.5 A g^−1^	196.15	[66]
Coconut fiber	H_3_PO_4_-assisted HTC	KOH	6 M KOH	1 A g^−1^	315.5	[15]
Coconut fiber	Hydrothermal treatment	KOH	1 M H_2_SO_4_	0.5 A g^−1^	332.1	This work

## Data Availability

The original contributions presented in this study are included in the article. Further inquiries can be directed to the corresponding authors.

## Abbreviations

The following abbreviations are used in this manuscript:

CF	Coconut Fiber
CF-AC	Coconut Fiber Activated Carbon
CF-OC-AC	Oxidatively Pretreated Coconut Fiber Activated Carbon
CF-HTC-AC	Hydrothermally Pretreated Coconut Fiber Activated Carbon
CV	Cyclic Voltammetry
GCD	Galvanostatic Charge–Discharge
EDLC	Electric Double-Layer Capacitance
PC	Pseudocapacitance
FTIR	Fourier Transform Infrared Spectroscopy
XRD	X-ray Diffraction
